# Research on Joint Parameter Inversion for an Integrated Underground Displacement 3D Measuring Sensor

**DOI:** 10.3390/s150408406

**Published:** 2015-04-13

**Authors:** Nanying Shentu, Guohua Qiu, Qing Li, Renyuan Tong, Nankai Shentu, Yanjie Wang

**Affiliations:** 1 College of Mechanical & Electrical Engineering, China Jiliang University, Hangzhou 310018, China; E-Mails: stnying_2@163.com (N.S.); tongrenyuan@126.com (R.T.); wangyanjiexx@163.com (Y.W.); 2 College of Information Engineering, China Jiliang University, Hangzhou 310018, China; E-Mail: qghfr@163.com; 3 National Engineering Research Center of Advanced Rolling, University of Science & Technology Beijing, Beijing 100083, China; E-Mail: stnk@nercar.ustb.edu.cn

**Keywords:** underground displacement, 3D measuring sensor, joint inversion of horizontal and vertical displacement, forward modeling, approximate optimization inversion

## Abstract

Underground displacement monitoring is a key means to monitor and evaluate geological disasters and geotechnical projects. There exist few practical instruments able to monitor subsurface horizontal and vertical displacements simultaneously due to monitoring invisibility and complexity. A novel underground displacement 3D measuring sensor had been proposed in our previous studies, and great efforts have been taken in the basic theoretical research of underground displacement sensing and measuring characteristics by virtue of modeling, simulation and experiments. This paper presents an innovative underground displacement joint inversion method by mixing a specific forward modeling approach with an approximate optimization inversion procedure. It can realize a joint inversion of underground horizontal displacement and vertical displacement for the proposed 3D sensor. Comparative studies have been conducted between the measured and inversed parameters of underground horizontal and vertical displacements under a variety of experimental and inverse conditions. The results showed that when experimentally measured horizontal displacements and vertical displacements are both varied within 0 ~ 30 mm, horizontal displacement and vertical displacement inversion discrepancies are generally less than 3 mm and 1 mm, respectively, under three kinds of simulated underground displacement monitoring circumstances. This implies that our proposed underground displacement joint inversion method is robust and efficient to predict the measuring values of underground horizontal and vertical displacements for the proposed sensor.

## 1. Introduction

The effective monitoring of underground displacement is an important means to prevent and mitigate such major geological hazards as landslide, collapse, debris flow and subsidence [1,2,3]. It is also a practical engineering tool to evaluate quality and risk for a wide range of hydraulic and geotechnical projects, including roads, railways, tunnels, dams, mining sites, and so on [4,5,6,7]. It can go deeply into the studied rock and soil mass to detect and measure the underground layered displacements and deformation quantity. For various kinds of geotechnical mass, underground displacement monitoring can effectively locate the sliding surfaces, determine the deformation mode, evaluate the deformation ranges and predict the deformation trend. Therefore, it can provide more objective and detailed information for deformation mechanics analysis, stability/safety assessment, hazard prediction/forecasting and prevention and mitigation project design [8,9,10]. However, due to the extremely variable and complex properties of monitored underground geotechnical mass, such as spatial invisibility, temporal randomness, environmental terribleness and geological heterogeneity, underground displacement monitoring technology was slowly developed. Most of the existing underground displacement monitoring methods, including inclinometers, settlement gauges, extensometers and TDR (time domain reflectometry), suffer from certain limitations, including low accuracy, high cost, narrow range, poor adaptability, low automation, poor long-term durability and susceptibility to electromagnetic interference [11,12,13,14,15]. In recent years, optical fiber sensing technologies have been rapidly developed for structural health monitoring of geotechnical infrastructures, such as bridges, dams and tunnels [16,17,18], and many exploratory research projects and experiments have been actively conducted for their application on the subsurface displacement monitoring of geotechnical mass [19,20]. Compared to the conventional underground displacement instrumentation, optical fiber sensors have specific merits, including tiny size, light weight, high precision, immunity to electromagnetic interference and resistance to corrosion. In the geotechnical domain, Ho [21] designed an FBG (fiber Bragg grating)-segmented deflectometer to measure the relative deflection segment by segment within the geotechnical mass concerned. Pei [22] developed an FBG-based in-place inclinometer to monitor the underground displacement of the Weijiagou Landslide in China. Zhu [23] developed a surface-adhered FBG sensing bar to monitor the internal displacements of a model gravity dam. Optical fiber sensor techniques have revealed application potential to underground displacement monitoring. However, some challenges still exist before their full practical application, including the fiber fragility, limited measuring range, low embedding survival rate and cross sensitivity of strain and temperature [24].

Thanks to our previous research [25,26], an electromagnetic underground displacement three-dimensional (3D) measuring sensor had been advocated on the basis of a unique array structure design of integrated sensing units and integration of various magnetoelectric effects, including electromagnetic induction, the Hall effect and the magnetoresistance (MR) effect. It was abbreviated as the electromagnetic underground displacement 3D sensor.

**Figure 1:**
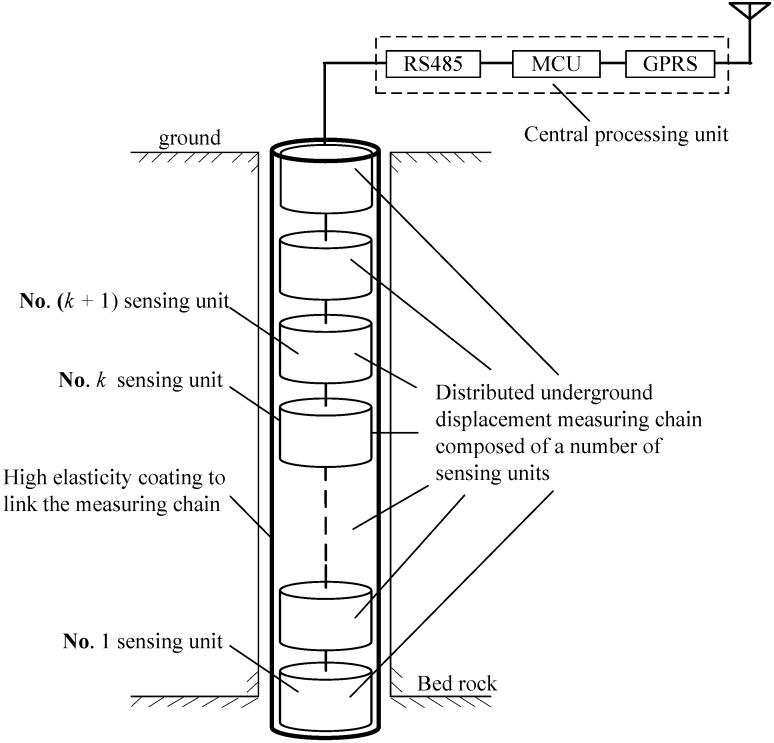
Schematic diagram of the electromagnetic underground 3D measuring sensor. GPRS, general packet radio service.

As Figure 1 shows, the proposed underground displacement 3D sensor is composed of a number of cylindrical electromagnetic sensing units and an information central processing unit. Each sensing unit has an identical structure, whose outer wall is an air-cored solenoid and inner wall is embedded with the integrated sensing PCB (printed circuit board). All sensing units are serially connected through the power lines, communication lines and signal lines and linked as a chain with high elastic connecting materials after epoxy resin coverage. The monitoring data of sensing units can be further transmitted to the remote computer through the GPRS (general packet radio service) wireless network for processing, display and prediction, thereby realizing a real-time measuring and monitoring of underground 3D displacements.

During the actual installation process, all of the sensing units are vertically buried into a drilling hole of equidistance and backfilled tightly. The coordinates of the top (surface) sensing unit are determined by GPS or mapping standard point coordinates. Along with the sliding and deformation of surrounding rock and soil mass, the relative displacement and inclination may occur between any two adjacent sensing units. According to the electromagnetic induction and Hall effects, the output values of mutual inductance *U*_o_ and Hall voltage *U_H_* between two adjacent units will be changed accordingly. Meanwhile, the sensor’s built-in integrated tilt measuring module can measure the relative tilt angle *θ*_0_ between them. By implementing a certain measurement algorithm, the simultaneous variations of *U*_o_, U_H_ and *θ*_0_ can be converted to the measuring values of relative horizontal displacement, vertical displacement and tilt angle between two corresponding adjacent sensing units. During the whole measuring process, under the centralized control of the information processing unit, the relative horizontal displacements, vertical displacements and tilt angles between two adjacent units can be successively measured from bottom to top. Therefore, the sensor can realize an automatic, real-time and complete monitoring and measuring of underground horizontal displacements, vertical displacements and tilt angles from the surface to different depths (till to the bedrock) within the detected underground mass. 

Compared to the existing underground displacement monitoring instrumentations, our proposed underground displacement 3D measuring sensor has such sensing properties as follows

(1) Simple structure, convenient manufacturing and relatively low cost.
(2) Relatively convenient installation and good portability thanks to its measuring chain assembly.
(3) Big measuring range, quite high measuring and positing accuracy.
(4) Good consistency between the measured and the actual deformation of geotechnical mass, thanks to the flexible structure of the sensing unit measuring array, where each sensing unit can freely move and deform accompanying the movement of the surrounding geotechnical mass.
(5) Three-dimensional measurement of the geotechnical underground displacement, rather than one-dimensional measurement by most of the existing instruments.

For the proposed sensor, in order to achieve an automatic and accurate measurement toward the underground 3D displacements, not only high quality design and manufacturing are required, but also an in-depth theoretical research on the underground displacement sensing mechanism and measuring property is urgently needed. Among them, how to establish some precise and efficient underground displacement parameter measuring models and parameter inversion methods is of vital importance. 

In our previous works, thanks to comprehensive studies on various factors and parameters affecting the proposed sensor’s sensing characteristics, two underground displacement measuring models with such virtues as sound estimation accuracy, high computation efficiency and easy hardware implementation were advocated. They are the NIELA (numerical integration-based equivalent loop approach) mutual inductance voltage measuring model [26] and EMC-NI (equivalent magnetic charge-numerical integration model) Hall voltage measuring model [27]. These two models can quite accurately evaluate the complicated relationship among the proposed 3D sensor’s varied output of mutual inductance voltage (*U*_o_) and Hall voltage (*U*_H_), respectively, the measuring parameters—The relative horizontal displacement, vertical displacement and tilt angle between any two adjacent sensing units—And the shape, geometry and material property parameters of the sensing units. The measuring parameters can represent the relative horizontal displacement, vertical displacement and tilt angle at different underground depths, namely the different buried depths of sensing units within the monitored geological mass.

However, the monitoring objects of the underground displacement 3D sensor are mainly the complex, variable and invisible subsurface rock and soil mass with complicated nonlinear characteristics. The sensor’s output are not directly measuring underground displacements and sliding angles, but the mutual inductance voltage and Hall voltage, short of physical meaning. Moreover, these two mentioned measuring models are quite abstract and complex. Therefore, a focal point should be taken when conducting the fundamental research of underground displacement measurement theory and methods, namely the study of the underground displacement parameter inversion approach for the proposed sensor. This implies how to make full use of the previously presented NIELA and EMC-NI models as an underground displacement measuring theoretical basis to establish some reliable and efficient underground displacement inversion approaches, to convert the real-time output of mutual inductance voltage and Hall voltage into the sensor’s measuring parameters—Underground horizontal displacement, vertical displacement and tilt angle at different depths within rock and soil mass—Directly. 

In this paper, an innovative displacement joint inversion method coupling innovative forward modeling and approximate optimization inversion has been proposed. It applies the semi-analysis NIELA and EMC-NI models as the join forward models to generate the reference signals of mutual inductance voltage and Hall voltage simultaneously, which combined with the measured signals and some related parameters, are input into the proposed inversion system. Through further execution of the joint optimization algorithm, the inversion system can finally realize joint inversions of the measuring underground horizontal displacement and vertical displacement parameters for the proposed 3D sensor with fairly high prediction precision and efficiency.

## 2. Sensor Working Principle and Theoretical Modeling

**Figure 2:**
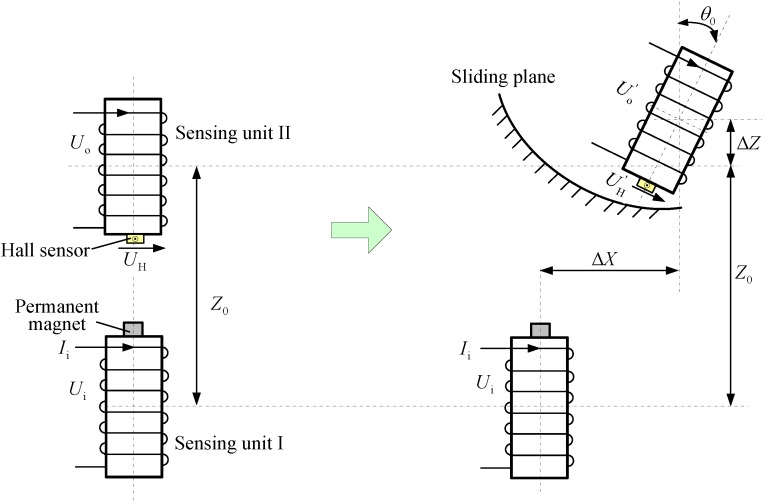
Principle diagram of the proposed underground displacement 3D sensor (only two adjacent sensing units are given). (**a**) Initial geometrical arrangement; (**b**) appearances of relative horizontal displacement Δ*X*, vertical displacement Δ*Z* and tilt angle *θ*_0_.

As shown in Figure 2, our designed electromagnetic underground displacement 3D measuring sensor is mainly worked on the following principle: driven by the movement of surrounding rock and soil mass, a relative horizontal displacement Δ*X*, vertical displacement Δ*Z* and tilt angle *θ*_0_ might synchronously occur between any two adjacent sensing units (referred to as Sensing Units I and II). Due to the electromagnetic induction and Hall effect, the mutual inductance voltage *U*_o_ and Hall voltage *U*_H_ generated between Sensing Units I and II will be varied simultaneously. Meanwhile, the relative axially tilt angle *θ*_0_ between them can be automatically measured by the built-in tilt measuring integrated module. Therefore, by virtue of the establishment of underground displacement measurement relationship models and GPS-based ground coordinate measurement, the proposed sensor can realize sequentially from bottom to top the measurement of Δ*X*, Δ*Z* and *θ*_0_ at different depths within the monitored mass and convert them into the underground 3D deformation coordinates consistent with the GPS space coordinates. It should be stated that the proposed underground displacement measurement relationship models can effectively describe the complex relationship among the variations of *U*_o_, *U*_H_ and *θ*_0_ between any two adjacent sensing units, the measuring parameters (relative horizontal displacement Δ*X*, vertical displacement Δ*Z* and tilt angle *θ*_0_ at corresponding underground depths) and the geometry and property parameters of the sensing units. The geometry and property parameters mainly include the length, diameter and winding coil turns of the sensing units, the shape, size and magnetization characteristics of the permanent magnet, the sensing characteristics of the Hall sensor and the I/O relationship of the Hall voltage measuring circuitry.

**Figure 3:**
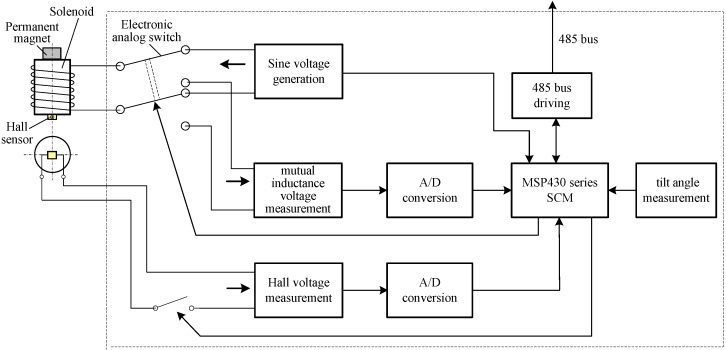
Sensing unit component diagram.

Each sensing unit has the same structure. As shown in Figure 3, its outer wall is an air-cored cylindrical solenoid, and the inner wall is wedged with several integrated sensing PCB combining such function modules as the permanent magnet, Hall sensor, SCM (single-chip microcomputer), sine voltage generation, mutual inductance voltage measurement, Hall voltage measurement, tilt angle measurement, A/D conversion and RS485 communication.

In consideration of various factors affecting the sensing characteristics and after in-depth theoretical study of the proposed 3D sensor, we had established two quite efficient and accurate semi-analytic underground displacement measuring theoretical models: the NIELA-based mutual inductance voltage measuring model and the EMC-NI based Hall voltage measuring model. These two models have been detailed in our previously published papers [26,27]. Here, we only make a brief introduction.

The NIELA (numerical integration-based equivalent loop approach) mutual inductance voltage measuring model was established by technical fusion of the electromagnetic field theoretical study, equivalent loop modeling on solenoid and the numerical integration approach. As Figure 2 shows, for the proposed 3D sensor, it can qualitatively characterize the functional relationship among the varied outputs of mutual inductance voltage *U*_o_ between any two adjacent sensing units, the measuring parameters between them and the geometrical and property parameters of sensing units. Among them, the measuring parameters include the relative horizontal displacement Δ*X*, relative vertical displacement Δ*Z* and relative tilt angle *θ*_0_. The geometrical and property parameters mainly include the solenoids’ length *h*, diameter *d*, number of windings *w* and the initial vertical distance *Z*_0_ and horizontal distance *X*_0_ between them. Generally speaking, NIELA is essentially a semi-analytic calculation approach with quite high accuracy.

Through the comprehensive application of Hall sensing mechanism analysis, 3D spatial distribution modeling to the magnetic field of the permanent magnet and the multidimensional numerical calculation method, the EMC-NI (equivalent magnetic charge-numerical integration approach) was proposed and served as the proposed sensor’s Hall voltage measuring model. It provides a quite precise and efficient description of the functional relationship among the sensor’s Hall voltage output *U*_H_ between Sensing Units I and II, the measuring parameters Δ*X*, Δ*Z* and *θ*_0_ between them and the geometrical and sensing properties of the sensing units and permanent magnet.

## 3. Underground Displacement Parameter Joint Inversion Method

The built-in circuitry modules in the proposed 3D sensor cannot directly output the measuring underground horizontal displacement and vertical displacement, but the relatively abstract physical quantity: the mutual inductance voltage and Hall voltage. Therefore, another important research content of underground displacement measurement is how to make further usage of the above theoretical modeling results to work out some quite efficient and practical underground displacement parameter inversion approaches. The purpose is to inversely deduce the measuring underground horizontal and vertical displacement parameters at different underground depths (the axial tilt angle *θ*_0_ can be directly measured by the sensor circuitry) from the synchronous output variables of mutual inductance voltage and Hall voltage between Sensing Units I and II. 

Generally speaking, the parameter inversion method is an effective way to inversely predict one or more initial parameters (such as speed, displacement, initial stress or geometric parameters) through minimizing the differences between the monitoring data and the modeling calculation results [28]. It is characterized by intensively studying some field measurable physical parameters (e.g., load, stress and strain) capable of describing the system behaviors and establishing some reasonable mathematical or physical back-analysis models to simulate the unknown practical system. Then, it implements the effective parameter adjustment process to modify the model parameter gradually, so as to minimize the error (difference) between the inversion calculation results and the system actual measurements [29,30]. An effective parameter inverse procedure at least consists of three elements [31]:

(1) Data collected from the unknown system should be as precise and timely as possible;
(2) The calculation model to describe the unknown system should be sensible and representative;
(3) The parameter adjustment algorithm must be efficient and convergent.

Presently, the parameter inversion methods are mainly developed along two ways: (1) some evolutionary inversion methods are developed mainly aimed at raising the inversion theoretical depth or improving the inversion scheme, such as genetic algorithm, artificial neural network, ant colony algorithm, evolution algorithm and simulated annealing [32,33,34,35]; and (2) some practical and simple inversion approaches are put forward mainly targeted at solving some practical geo-engineering problems [36,37].

According to the practical engineering requirements of underground displacement monitoring, this paper proposes a simple and efficient inversion approach of underground displacement parameters with quite high prediction accuracy and efficiency. It is called the “joint forward simulation-optimization inversion method”. It is aimed at solving the simultaneous and direct measurement problem of underground horizontal, vertical displacement and tilt angle for the proposed 3D sensor.

As mentioned, it is very important to establish some representative and accurate calculation models to simulate the unknown system. For our proposed sensor, by virtue of previous studies, two high efficiency and approximate underground displacement measuring theoretical models have been established, namely the NIELA and EMC-NI models, to describe the complex relationships among the sensor’s output of mutual inductance voltage and Hall voltage, respectively, its measuring parameters—Underground horizontal displacement, vertical displacement and tilt angle—And the sensor’s geometry and sensing property parameters. Thereby, according to the practical requirement of underground displacement monitoring and measurement, firstly, this paper combines the NIELA model with the EMC-NI model to establish the parameter inversion mathematical models, which are called the joint forward simulation models of underground displacement. Secondly, the initial model parameters and trial estimates of inversing parameters are input into the NIELA and EMC-NI forward simulation models simultaneously, and after a run of the execution program of forward simulation, output sequences of the simulated (theoretical) values of the mutual inductance voltage and Hall voltage are obtained simultaneously. This process is called the “joint forward simulation process”. Based on these, integration of NIELA and EMC-NI forward simulation models with the combined optimization inversion algorithm makes up our proposing joint parameter inversion method, which can realize a simultaneous inversion of underground horizontal displacement and vertical displacement for the proposed 3D sensor. 

Here, we give a brief introduction to the optimization inversion procedure. It is a direct parameter inverse analysis approach following the following optimal control principle:

Let a point in *l-*dimensional space be depicted as *h* = (*p*_1_, *p*_2_, …, *p_l_*); then, the point collection satisfying *m_i_* < *p_i_* < *n_i_* constitutes a specific domain in the *l*-dimensional space and may be marked as *p*. If the value of target function $J(\overset{-}{p})$ is set as the standard to measure whether {*p*} accords with the actual requirement, the parameter inversion problem converts to an optimization search problem, namely to solve {*p*} when satisfying:

(1) $$\begin{array}{cc} J(\overset{-}{p})=\underset{\overset{-}{p}\in P}{\min}E(p), & \;\;\;\;\overset{-}{p}\in P \end{array}$$

The solutions to such kinds of problems are called the optimal control methods, which usually resort to the iterative process for solution. First, set a group of initial estimate values for the parameters to be inversed (predicted). Next, repeatedly execute the iterative process and revise these parameters according to the iteration results or feedbacks step by step, so that the value of objective function *J* (such as the minimum error function) can be gradually decreased under certain restrictive conditions. Then, the optimized values are tested with the specified convergence criteria. Once the convergence conditions are satisfied, the optimization process ends, and the final iteration results are output as parameter inversion values.

**Figure 4:**
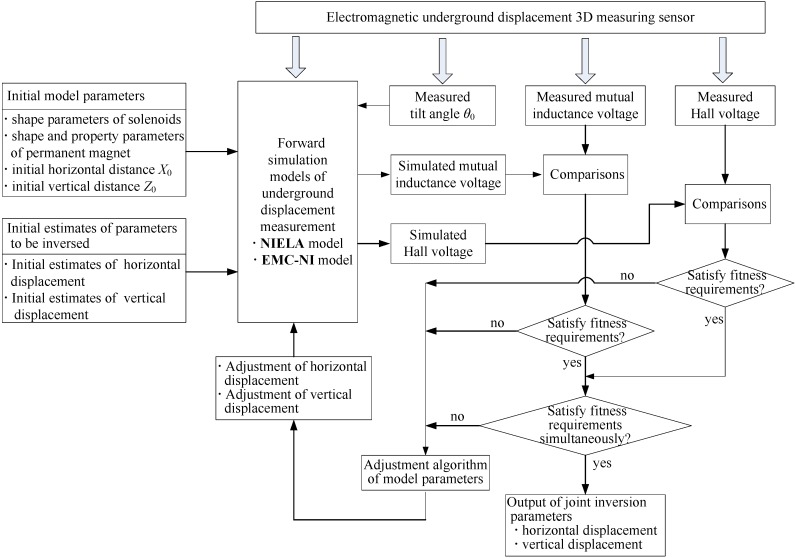
Schematic diagram of the displacement parameter joint inversion method. NIELA, numerical integration-based equivalent loop approach; EMC-NI, equivalent magnetic charge-numerical integration.

Figure 4 shows a schematic diagram of the “joint forward simulation-optimization inversion method” for the proposed 3D sensor. The inversion process mainly includes three steps:

(1) Data acquisition of mutual inductance voltage *U*_o_, Hall voltage *U*_H_ and tilt angle *θ*_0_ for the proposed sensor. The data may come from two ways: Usually, it is the on-site measuring results when the sensor has been buried into the monitored rock and soil masses for months or years. Sometimes, it can be the experimental measurement values for sensor testing and research purposes, where the values of relative displacement and tilt angle (Δ*X*, Δ*Z*, *θ*_0_) between two adjacent sensing units are artificially varied, so as to measure and record the synchronous variations of *U*_o_ and *U*_H_. In this paper, the latter way is adopted.
(2) Execution of the NIELA and EMC-NI joint forward simulation process. The initial model parameters and the initial estimate values of relative horizontal displacement and vertical displacement are fed into the execution programs of the forward simulation models. Therefore, the simulated values of mutual inductance voltage and Hall voltage can be generated. The initial model parameters include the geometrical and property parameters of the solenoids and permanent magnets.
(3) Execution of the joint optimization inverse process. Make a combined comparison between the simulated values of the mutual inductance voltage and Hall voltage obtained in Step (2) with the corresponding measured values in Step (1) and gradually adjust the relative horizontal displacement and vertical displacement values by the joint parameters modification algorithm. This process continues until not only the fitness between the simulated and the measured mutual inductance voltage can meet the required accuracy (or gets the minimum discrepancy under certain conditions), but also the fitness between the simulated and measured Hall voltage achieves optimality under specified constrains. The final iterative results are then output as the joint inversion results of horizontal displacement Δ*X* and vertical displacement Δ*Z* for the proposed sensor. They can be treated as the measuring values of relative horizontal displacement and vertical displacement at some underground depth where the corresponding sensing units are buried. The iterative values of Δ*X* and Δ*Z* combined with the measured value of relative tilt angle *θ*_0_ constitute the complete monitoring results at the corresponding subsurface depth within the monitored geological mass for the proposed 3D sensor.

## 4. Experiments and Verifications of Underground Displacement Joint Inversion 

To verify the above proposed underground displacement parameter joint inversion method, a series of experiments of underground horizontal and vertical displacement joint inversion will be conducted for the proposed underground displacement 3D sensor in this section. 

### 4.1. Experiment Setup and Procedure

The sensor’s output quantities include the mutual inductance voltage *U*_o_, Hall voltage *U*_H_ and relative axial tilt angle *θ*_0_ between any two adjacent sensing units, and the parameters to be inversed are the relative horizontal displacement and vertical displacement between them, responding to the sensors’ measuring underground horizontal displacement Δ*X* and vertical displacement Δ*Z* at some given underground depth. Below, the related parameter inversion experiment setup and evaluation scheme are briefly introduced.

#### 4.1.1. Experiment Equipment

**Figure 5:**
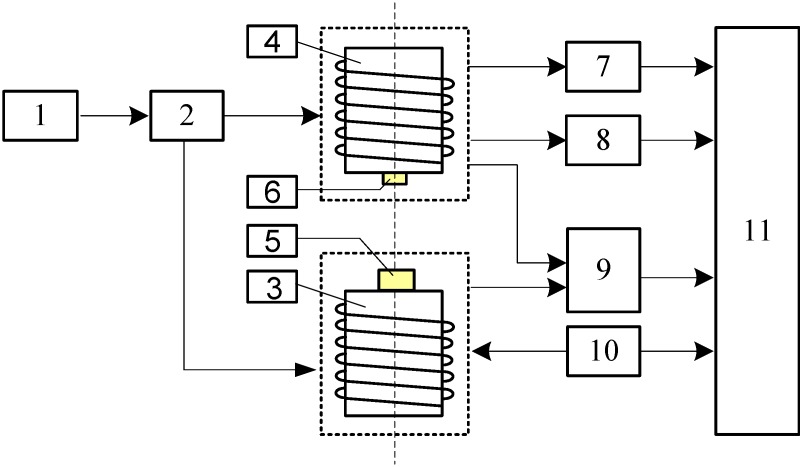
Schematic diagram of the experimental setup. 1, 4-axial driving controller; 2, 4-axial driving unit; 3, Solenoid I; 4, Solenoid II; 5, permanent magnet; 6, Hall sensor; 7, mutual inductance voltage measuring circuit; 8, Hall voltage measuring circuit; 9, tilt measuring circuit; 10, sine voltage generating circuit; 11, single-chip microcomputer (SCM) system.

Parameter inversion experiments are conducted on the electromagnetic underground displacement 3D measurement experimental platform, which was constructed at China Jiliang University’s Geological Disaster Monitoring Equipment Research Center and had been detailed in our previous works [26,27]. Here, we only briefly describe it. As shown in Figure 5, the experimental platform mainly includes three parts: (1) prototype of the electromagnetic underground displacement 3D measuring sensor, mainly composed of two adjacent integrated sensing units, referred to as Sensing Units I and II; (2) four-axis drive controller and device for underground displacement measurement centered by PC and stepper motors. They can continuously adjust the relative horizontal displacement Δ*X*, vertical displacement Δ*Z* and tilt angle *θ*_0_ between two sensing units with an adjustment precision of a 0.2° axial angle and a 0.1-mm displacement, respectively; (3) SCM control system; this can implement an automatic measurement and real-time recording of such measuring parameters as the relative horizontal Δ*X*, vertical displacement Δ*Z* and axial tilt angle *θ*_0_ between Solenoid I and II and the sensor’s output of mutual inductance voltage *U*_o_ and Hall voltage *U*_H_.

#### 4.1.2. Experiment and Evaluation Scheme

The horizontal and vertical displacement joint inversion experiments and evaluation are as follows:

(1) Before the experiment, the initial vertical distance *Z*_0_ and axial tilt angle *θ*_0_ for the proposed sensor are set as fixed values, and the initial horizontal distance *X*_0_ is set as zero. During the experiment, we change point-by-point the sensor’s relative horizontal displacement *∆X_i_* (*i* = 1, 2, …, m) and vertical displacement *∆Z_j_* (*j* = 1, 2, …, *n*) and record the synchronous output values of the mutual inductance voltage *U*_o*ij*_ and Hall voltage *U*_H*ij*_, thus forming the variation sequences of measured mutual inductance voltage *U*_o_ = {*U*_o11_, *U*_o12_, …, *U*_omn_} and Hall voltage *U*_H_ = {*U*_H11_, *U*_H12_, …, *U*_Hmn_} required by the parameter inversion process. Meanwhile, these two sets of measured horizontal displacement and vertical displacement variable series *∆X* = {*∆X*_1_, *∆X*_2_, …, *∆X*_m_} and *∆Z* = {*∆Z*_1_, *∆Z*_2_, …, *∆Z*_m_} can be treated as the true (measured) values of horizontal displacement and vertical displacement needing inversing for the proposed sensor.
(2) Both the initial model parameters and the trial estimate sequences of horizontal displacement and vertical displacement (*ΔX'* and *ΔZ'*) are input into the NIEAL and EMC-NI joint forward simulation models. The initial model parameters include two parts: (i) shape parameters, mainly composed of the diameter *d* = 75 mm, length *a* = 70 mm, coil turns *w* = 400 of Solenoid I and II, the diameter *D* = 7.5 mm and height *H* = 18 mm of the permanent magnet; (ii) initial geometric position parameters, representing by *Z*_0_, *X*_0_ and *θ*_0_ and setting as the same values as in Step (1).
(3) Execution of the joint forward simulation-optimization inversion procedure on every point pair [*U*_o*ij*_, *U*_H*ij*_] of the measured sequences of mutual inductance voltage *U*_o*i*_ and Hall voltage *U*_H*ij*_, to figure out the inversed values of horizontal displacement ∆*X*'*_i_* and vertical displacement ∆*Z*'*_j_*; then arranging ∆*X*'*_i_* and ∆*Z*'*_j_* chronologically to form the complete inversed sequence of *∆X*' = {*∆X*'_1_, *∆X*'_2_, …, *∆X*'_m_} and *∆Z*' = {*∆Z*'_1_, *∆Z*'_2_, …, *∆Z*'_n_}.
(4) Making the deviation calculation and fitting analysis between the inversed series of horizontal displacement and vertical displacement in Step (3) with their corresponding measured series in Step (4), so as to testify to the suitability and stability of the proposed joint parameter inversion method.

### 4.2. Evaluation of Parameter Inversion Experiments 

The proposed sensor should perform a simultaneous monitoring of the underground horizontal displacement, vertical displacement and tilt angle at different underground depths within the monitored mass. Among them, the tilt angle *θ*_0_ can be real-time measured by the sensor’s tilt measuring integrated circuit, so it can be treated as a known output. Therefore, the sensor’s purpose of parameter inversion is to calculate the values of underground horizontal displacement and vertical displacement in reverse according to the sensor’s varied output of the mutual inductance voltage, Hall voltage and tilt angle. That is, when the tilt angle between two adjacent sensing units is known, the variations of the mutual inductance voltage and Hall voltage may be caused by three possibilities: the change of the horizontal displacement, the change of the vertical displacement and synchronous changes of the horizontal displacement and vertical displacement. For these three kinds of possible underground movement situations, we will apply the proposed “joint forward simulation-optimization inversion method” to conduct a series of underground horizontal and vertical displacement joint inversion experiments, so the prediction accuracy and robustness of our proposed parameter inversion method can be quantitatively evaluated. 

#### 4.2.1. Experiments and Parameter Inversion 1

First, we will conduct experiments and parameter inversions simulating the first kind of underground displacement monitoring possibility, where under the sliding impact of underground geotechnical engineering mass, the relative horizontal displacement between Sensing Units I and II keeps growing while the relative vertical displacement between them remains basically unchanged. Under this situation, the changes of the mutual inductance voltage and Hall voltage mainly result from variations of the underground horizontal displacement Δ*X* and tilt angle *θ*_0_.

**Figure 6:**
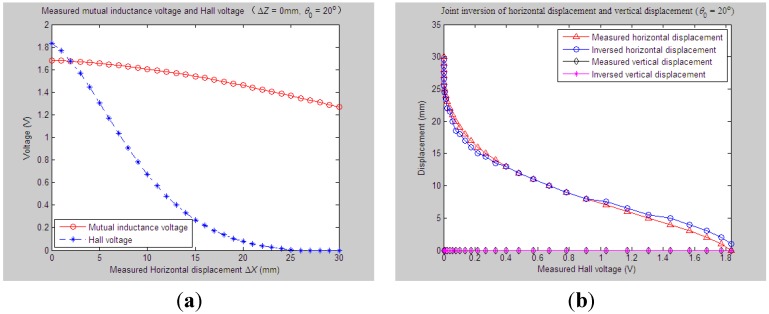
Horizontal and vertical displacement joint inversion results when measured Δ*X* are changed while the measured Δ*Z* remain stable (*θ*_0_ = 20°, Δ*Z* = 0 mm). (**a**) Measured mutual inductance voltage and Hall voltage; (**b**) inversed horizontal and vertical displacements.

**Figure 7:**
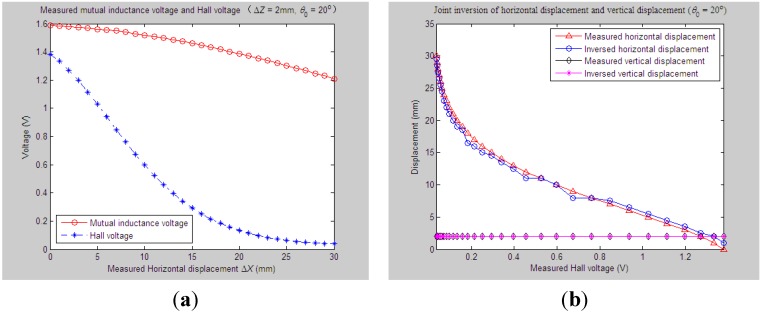
Horizontal and vertical displacement joint inversion results when measured Δ*X* are changed, while measured Δ*Z* remain stable (*θ*_0_ = 20°, Δ*Z* = 2 mm). (**a**) Measured mutual inductance voltage and Hall voltage; (**b**) inversed horizontal and vertical displacements.

**Figure 8:**
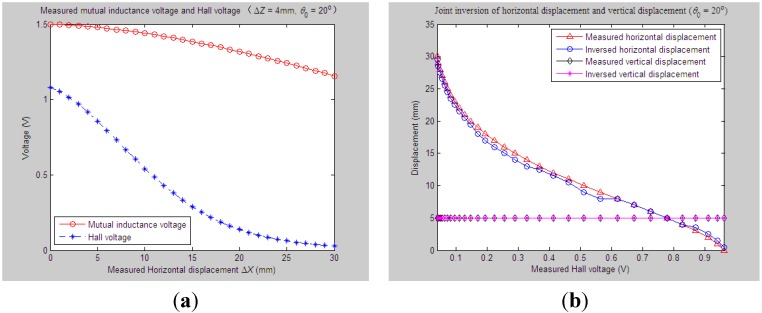
Horizontal and vertical displacement joint inversion results when measured Δ*X* are changed, while measured Δ*Z* remain stable (*θ*_0_ = 20°, Δ*Z* = 4 mm). (**a**) Measured mutual inductance voltage and Hall voltage; (**b**) inversed horizontal and vertical displacements.

**Figure 9:**
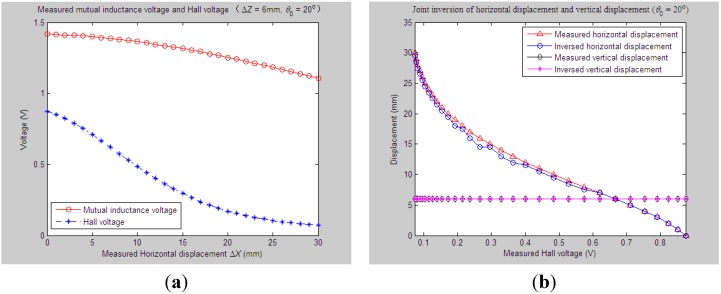
Horizontal and vertical displacement joint inversion results when measured Δ*X* are changed, while measured Δ*Z* remain stable (*θ*_0_ = 20°, Δ*Z* = 6 mm). (**a**) Measured mutual inductance voltage and Hall voltage; (**b**) inversed horizontal and vertical displacements.

**Figure 10:**
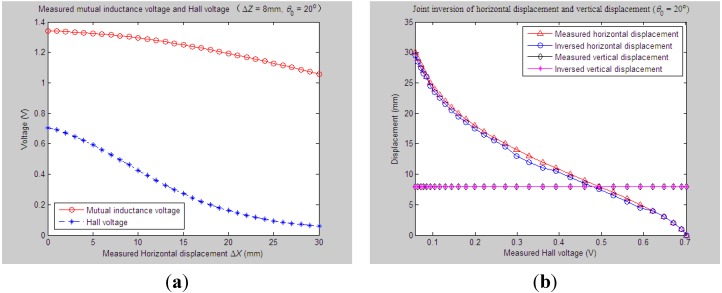
Horizontal and vertical displacement joint inversion results when measured Δ*X* are changed, while measured Δ*Z* remain stable (*θ*_0_ = 20°, Δ*Z* = 8 mm). (**a**) Measured mutual inductance voltage and Hall voltage; (**b**) inversed horizontal and vertical displacements.

According to experiment Step (1), we first set the initial values of vertical distance, horizontal distance and axial tilt angle between Sensing Units I and II as *Z*_0_ = 33 mm, *X*_0_ = 0 mm and *θ*_0_ = 20°, respectively. When the measured relative horizontal displacement Δ*X* successively varies from 0 to 30 mm, while the measured relative vertical displacement Δ*Z* basically remains at some stable values, such as 0 mm, 2 mm, 4 mm, 6 mm or 8 mm, the corresponding output of the mutual inductance voltage and Hall voltage are shown in Figure 6a, Figure 7a, Figure 8a, Figure 9a and Figure 10a. Figure 6b, Figure 7b, Figure 8b, Figure 9b and Figure 10b plot the joint inversion results of horizontal displacement Δ*X* and vertical displacement Δ*Z* after applying the proposed inversion method on the measured data of the mutual inductance voltages and Hall voltages when specifying the measured Δ*Z* to be 0 mm, 2 mm, …, and 8 mm, respectively. 

After a comprehensive analysis of the above figures, it can be seen that during the process of the measured Δ*Z* varied from 0 mm to 8 mm with a 2-mm interval, the inversion of vertical displacement is accurate, and the inversion deviation is less than or equal to 0.5 mm. Meanwhile, although the horizontal displacement inversion curves are not as smooth as the vertical displacement curves, the maximum inversion deviation is less than 3 mm and the average inversion deviation less than 1 mm. There exist only small overall deviations between the inversed and measured horizontal displacement. These experimental and inversion results preliminarily show that this is quite accurate and reliable to apply the proposed inversion method to the underground horizontal and vertical displacement joint inversion (prediction) for the proposed 3D sensor under conditions where the measured *ΔX* are actively changed and the measured Δ*Z* remain stable.

**Figure 11:**
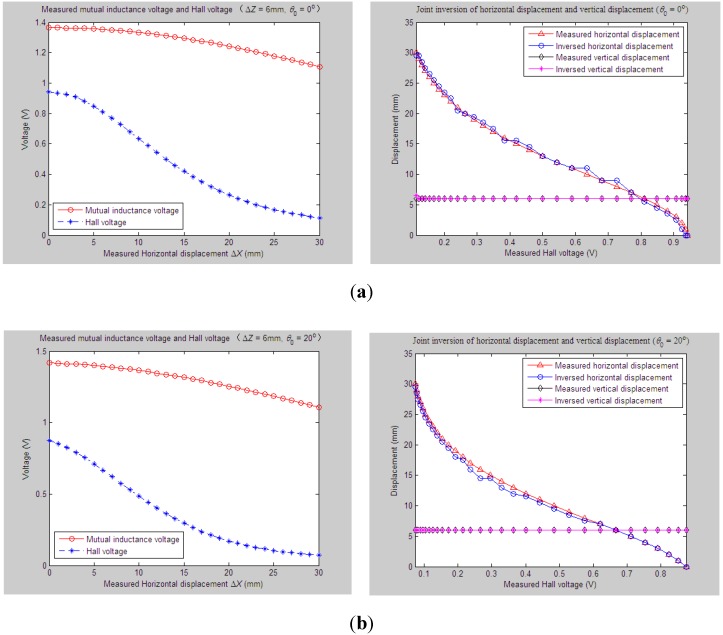

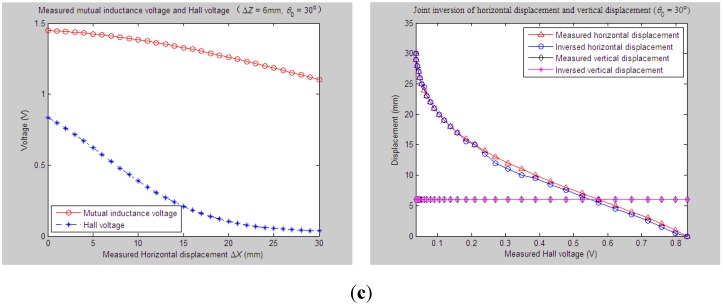
Inversed horizontal and vertical displacements *vs.* variations of Δ*X* and *θ*_0_ (Δ*Z* = 6 mm). (**a**) *θ*_0_ = 0°; (**b**) *θ*_0_ = 20°; (**c**) *θ*_0_ = 30°.

To further evaluate the validity and precision of the proposed joint parameter inversion method, we have inversed more horizontal displacement and vertical displacement values under more measured values of tilt angle *θ*_0_ (such as 0°, 10°, …, 50°) and vertical displacement Δ*Z* (such as, 0 mm, 2 mm …, 20 mm). As an example, Figure 11a–c displays the sensor’s joint inversion curves of horizontal displacement and vertical displacement when fixing *θ*_0_ to 0°, 20° and 30°, respectively, and setting the measured Δ*Z* to 6 mm, while the measured Δ*X* varied from 0 to 30 mm. For the above experiments and inversions conducted, some similar conclusions can be drawn: along with the simultaneous variations of measured *θ*_0_ and Δ*Z*, the inversed vertical displacements always show a stable and accurate tracking of the measured one, and the inversion deviations between them are less than 1 mm. Meanwhile, fairly good shape consistency and amplitude approximation have been achieved between the measured and simulated curves of horizontal displacements. The maximum and average inversion deviation of Δ*X* are controlled within 1 mm and 3 mm, respectively. This further validates that our proposed joint parameter inversion method is quite qualified to predict the measured underground horizontal and vertical displacement simultaneously for the proposed 3D sensor under the monitoring circumstance whose main underground deformation is the horizontal movement and inclination, while the vertical movement is negligible.

#### 4.2.2. Experiments and Parameter Inversion 2

Now, we will check the adaptability of the proposed inversion method to inverse underground horizontal and vertical displacements under the second monitoring conditions, such as ground settlement and mining subsidence, where the main forms of deformation are the vertical displacement and sliding.

**Figure 12:**
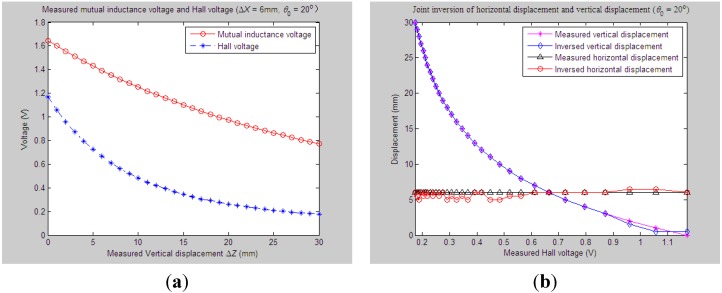
Horizontal and vertical displacement joint inversion results when measured Δ*Z* are changed while measured Δ*X* remain stable (*θ*_0_ = 20°, Δ*X* = 6 mm). (**a**) Measured mutual inductance voltage and Hall voltage; (**b**) inversed horizontal and vertical displacements.

**Figure 13:**
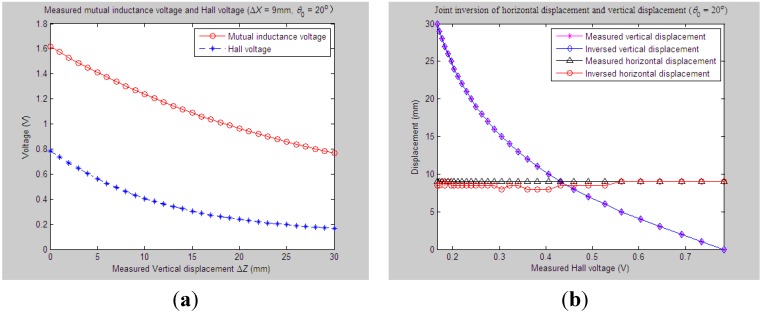
Horizontal and vertical displacement joint inversion results when measured Δ*Z* are changed, while measured Δ*X* remain stable (*θ*_0_ = 20°, Δ*X* = 9 mm). (**a**) Measured mutual inductance voltage and Hall voltage; (**b**) inversed horizontal and vertical displacements.

**Figure 14:**
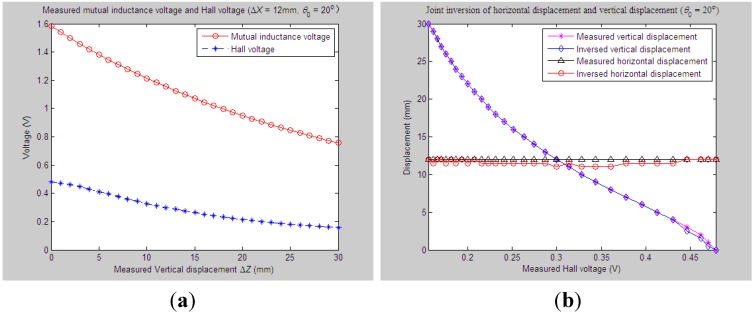
Horizontal and vertical displacement joint inversion results when measured Δ*Z* are changed, while measured Δ*X* remain stable (*θ*_0_ = 20°, Δ*X* = 12 mm). (**a**) Measured mutual inductance voltage and Hall voltage; (**b**) inversed horizontal and vertical displacements.

**Figure 15:**
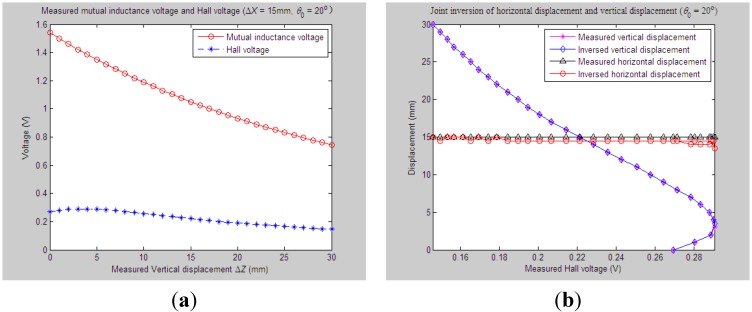
Horizontal and vertical displacement joint inversion results when measured Δ*Z* are changed, while measured Δ*X* remain stable (*θ*_0_ = 20°, Δ*X* = 15 mm). (**a**) Measured mutual inductance voltage and Hall voltage; (**b**) inversed horizontal and vertical displacements.

Similarly, for the proposed 3D sensor prototype, we first adjust the initial vertical distance *Z*_0_, horizontal distance *X*_0_ and tilt angle *θ*_0_ between Sensing Units I and II to be 33 mm, 0 mm and 20°, respectively. The measured relative vertical displacement Δ*Z* is dynamically varied from 0 to 30 mm, while the measured relative horizontal displacement Δ*X* is set as some constant values, including 6 mm, 9 mm, 12 mm and 15 mm, respectively. 2D graphs of the measured values of *U*_o_ and *U*_H_ *vs.* variations of Δ*Z* are demonstrated in Figure 12a, Figure 13a, Figure 14a and Figure 15a accordingly. For these measured voltage data, we apply the proposed inversion method to synchronously measure vertical displacement and horizontal displacement inversely, as shown in Figure 12b, Figure 13b, Figure 14b and Figure 15b.

As can be found, when the measured horizontal displacements are set as the above values, the vertical displacements can still be accurately inversed with a deviation of no more than 0.5 mm (measured Δ*Z* varied within 0 ~ 30 mm). Although the horizontal displacement inversed curves cannot highly coincide with the measured curves at some points, they usually stably fluctuate along the measured one with a fluctuation amplitude less than 3 mm and the inversion deviation not exceeding 2.5 mm.

Combined with more detailed parameter inversion and experiment results carried out by specifying more measured values of *θ*_0_ and Δ*X*, while the measured Δ*Z* continuously changes, some common conclusions can be drawn: (1) during the above inversion and experimental process, the inversion of vertical displacement remains stable and accurate and is little affected by variations of the measured tilt angle and horizontal displacement. The inversion curves of vertical displacement remain smooth and highly fit the measured curve with an inversion deviation no more than 1 mm; (2) The deviation accuracy of the horizontal displacement is lower than the vertical displacement to some degree due to its lower sensitivity. The horizontal displacement inversion curves are not quite smooth and often fluctuate slightly around the measured one with an inversion deviation of no more than 3 mm. The inversion of horizontal displacement can still meet sound fitting and inversion requirements. 

On the whole, the proposed “joint forward simulation-optimization inversion method” is quite accurate and reliable to derive the measured underground horizontal and vertical displacements simultaneously for the proposed sensor under the second monitoring situations where the underground vertical displacement is varied dynamically while the horizontal displacement remains stable.

#### 4.2.3. Experiments and Parameter Inversion 3

Experiment and Parameter Inversions 1 and 2 have examined and verified the proposed parameter inversion method under such instances that a significant change of horizontal displacement or vertical displacement is occurring, respectively. Now, we will further evaluate its inversion capability under the third monitoring possibility, where both the underground horizontal displacement and vertical displacement vary dynamically and significantly.

**Figure 16:**
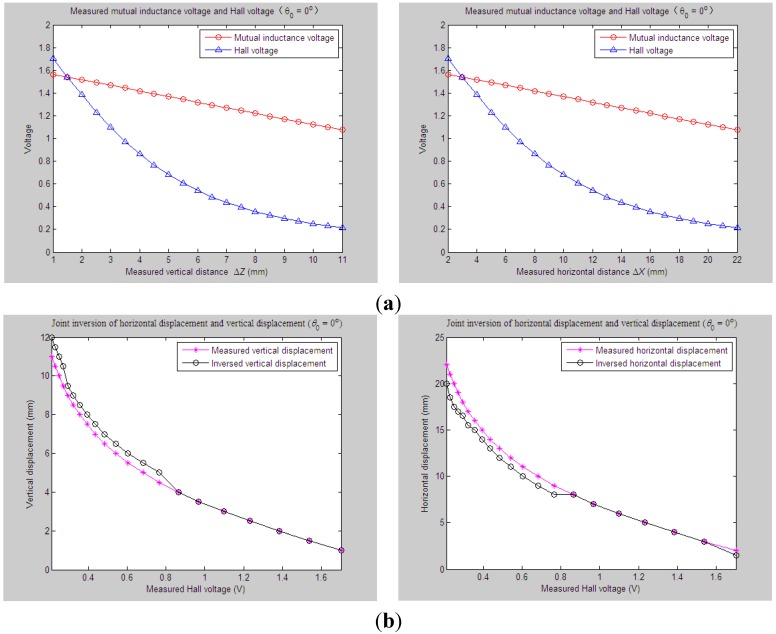
Horizontal and vertical displacement joint inversion results when both measured Δ*Z* and Δ*X* are changed (*θ*_0_ = 0°). (**a**) Measured mutual inductance voltage and Hall voltage; (**b**) inversed horizontal and vertical displacements.

Figure 16 shows the joint inversion results when applying the proposed inversion method on the experimentally measured mutual inductance voltages and Hall voltages, which are varied due to the synchronous occurrences of the horizontal displacement (Δ*X*) and vertical displacement (Δ*Z*) between Sensing Units I and II, while the tilt angle *θ*_0_ between them is supposed to remain at 0°. We can see that during the process of measuring, Δ*X* increases from 2 mm till 22 mm at 1-mm incremental steps; the measured Δ*Z* changes from 1 mm to 11 mm at a 0.5-mm interval synchronously. Corresponding to this, the output sequences of the mutual inductance voltage and Hall voltage [*U*_o_, *U*_H_] are changed from [1.56 V, 1.70 V] to [1.08 V, 0.21 V] gradually. Implement the joint parameter inversion method on these measured sequences of [*U*_o_, *U*_H_] to figure out the joint inversion values and deviations for measuring vertical displacement and horizontal displacement. The data analysis results show that the maximal and average inversion deviations of Δ*Z* are 1 mm and 0.43 mm, respectively, and the maximal and average deviations of Δ*X* reach 2.5 mm and 0.98 mm, respectively.

**Figure 17:**
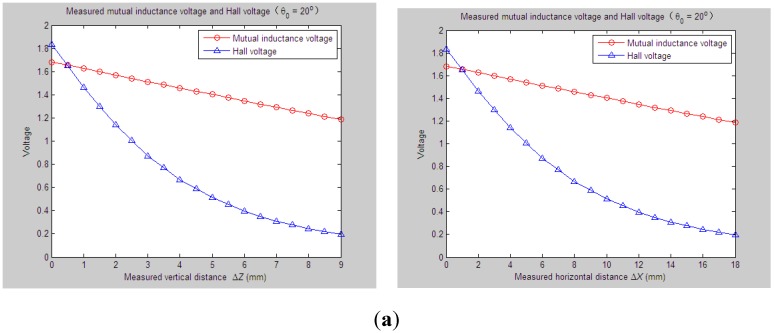

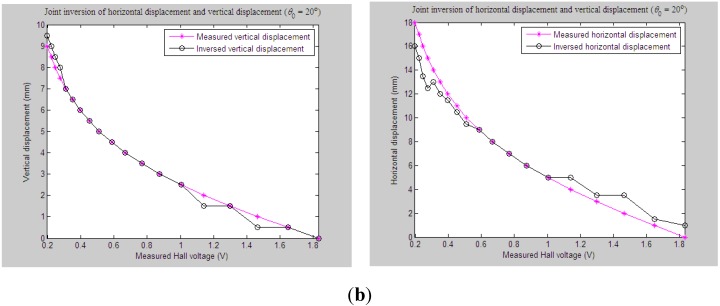
Horizontal and vertical displacement joint inversion results when both measured Δ*Z* and Δ*X* are changed (*θ*_0_ = 20°). (**a**) Measured mutual inductance voltage and Hall voltage; (**b**) inversed horizontal and vertical displacements.

Similarly, we can figure out the joint parameter inversion results of Δ*X* and Δ*Z* when the tilt angle *θ*_0_ is varied accompanied by simultaneous variations of measured Δ*X* and Δ*Z*. For example, when the measured Δ*X* and Δ*Z* are synchronously changed from 0 mm to 18 mm, 0 mm to 9 mm, respectively, while *θ*_0_ increases from 0° to 20°, the maximum and average inversion deviations of Δ*Z* are calculated as 0.5 mm and 0.16 mm, respectively, and the maximum and average inversion deviations of Δ*X* as 2.5 mm and 0.89 mm, respectively, as shown in Figure 17.

## 5. Conclusions

During the monitoring process of geological hazards and geotechnical engineering, it is often required to combine the monitoring of underground horizontal displacement and vertical displacement. However, due to the invisibility and complexity of underground displacement monitoring, there exist few practical underground displacement monitoring instruments or sensors that can monitor the subsurface horizontal and vertical displacements simultaneously.

In this paper, an innovative underground horizontal and vertical displacement joint inversion method referred to as the “joint forward simulation-optimization inversion method” has been presented. It can realize a joint inversion of underground horizontal displacement and vertical displacement for the proposed electromagnetic underground displacement 3D measuring sensor. A series of detailed comparisons, fitting analyses and deviation calculations between the measured and inversed parameters of horizontal displacement and vertical displacement have been carried out under various experimental conditions, where we set various values for the measured tilt angle *θ*_0_, horizontal displacement Δ*X* and vertical displacement Δ*Z* and varied some of them dynamically under three underground displacement monitoring circumstances. The results imply that our proposed joint parameter inversion method combining the specific NIELA and EMC-NI joint forward simulation with the optimization inversion procedure is quite efficient and robust to inverse horizontal displacement and vertical displacement simultaneously. The inversion results can quite accurately and stably predict the underground relative horizontal displacement and vertical displacement at different underground depths within the monitored geological/geotechnical mass. The deviations between the experimentally measured and modeling inversed Δ*X* and Δ*Z* were tested to be less than 3 mm and 1 mm, respectively.
